# Pulse Discovery Toolkit, a Multicomponent Nutrition Intervention for Preschool Children in Childcare Centers: Mixed Methods Pilot Study

**DOI:** 10.2196/55866

**Published:** 2026-05-22

**Authors:** Hiwot Abebe Haileslassie, Renee Ramikie, Hassan Vatanparast, D Dan Ramdath, Amanda Froehlich Chow, Phyllis Shand, Rachel Engler-Stringer, Jessica R L Lieffers, Shannon Hood-Niefer, Carol Henry

**Affiliations:** 1 College of Pharmacy and Nutrition, University of Saskatchewan, Saskatoon, SK, Canada; 2 Agricultural, Food and Nutritional Science, University of Alberta, 8602-112 St, Edmonton, AB, T6G 2E1, Canada, 1 780 492 7742; 3 School of Public Health, University of Saskatchewan, Saskatoon, Saskatoon, SK, Canada; 4 College of Agriculture and Bioresources, University of Saskatchewan, Saskatoon, SK, Canada; 5 Department of Community Health and Epidemiology, University of Saskatchewan, Saskatoon, SK, Canada; 6 Saskatchewan Food Industry Development Centre Inc, Saskatoon, SK, Canada

**Keywords:** childcare center, nutrition intervention, preschool children, pulse, sensory evaluation, pediatric, child, children, nutrition, nutritional, dietary

## Abstract

**Background:**

Children’s eating habits are formed at an early age, making childhood a crucial period for introducing novel foods, such as pulse-based food products. Pulse Discovery Toolkit (PDTK) intervention was designed to increase familiarity with pulses and to eventually contribute to the consumption of pulse-based foods among preschool children in childcare centers (CCs).

**Objective:**

To determine PDTK’s impact on knowledge, acceptability, and consumption of pulse-based foods among preschool children attending CCs, and to assess its feasibility and acceptability by early childhood educators (ECE) and cooks. The nutrient contents and food group servings of pulse-based intervention recipes in the PDTK were also compared with regular CC recipes.

**Method:**

The PDTK intervention was delivered over a 3-month period in 2 CCs in Saskatoon (50 children, 8 staff). The intervention, which integrated taste exposure and nutrition education, consisted of 12 child-friendly weekly lessons, a food service guide for cooks, 15 recipes for pulse-based foods, 4 intervention recipes incorporated in the CC menu, and 4 parent newsletters. Mixed methods were used with pre- and postintervention knowledge tests, plate waste measurement, sensory evaluation, ECE and cook’s perspective, and nutrient content comparison of the intervention and control foods from the regular childcare menu to evaluate the intervention’s impact.

**Result:**

Improvements in correct identification of chickpeas (2/21 [10%] at preintervention to 7/21 [33%] at postintervention, *P*=.074), beans (8/21 [38%] to 11/21 [52%], *P*=.68), and peas (6/21 [27%] to 8/21 [38%], *P*=.61) were not statistically significant. Children consumed higher amounts of the regular recipes (293.54, SD 27.65; 178.46, SD 24.33) than the intervention recipes (211.56, SD 25.61; 108.83, SD 21.97) at both times, respectively. However, at the end of the intervention, significant differences were only observed in the amount of total food consumption (*P*=.049) and the protein content (*P*=.04) when consumption proportion was examined, with both being higher for the control recipes in comparison to the intervention recipes. The majority (92% and 72%) of the children rated the refried bean wrap and lentil smoothie, “yummy,” respectively. Most of the intervention recipes have lower energy, fat, and sodium content compared with the regular CC recipes. Findings from ECE semistructured interviews and the lesson plan evaluations revealed that the ECEs reacted favorably to the curriculum. The cooks from the participating CCs did not report any barriers to cooking pulses in their facility. However, the need for modification to make the recipes easier to cook in CCs was noted in our study.

**Conclusions:**

With a few modifications to make some of the lessons more age-appropriate and some of the recipes easier to cook, it is feasible to implement the PDTK in CCs in order to promote regular consumption of pulses.

## Introduction

Children’s eating habits are formed at an early age, making the first 5 years of life a crucial period for introducing novel healthy foods. A growing body of evidence supports the need for repeated exposure to ensure liking and preference of novel foods [1-3]. Many researchers contend that childcare centers (CCs) are useful for the delivery of nutrition interventions as they provide continuous and intensive contact with children at an age when they develop life-long dietary preferences and eating patterns [4]. The fact that children often consume about 70% of their daily nutrient intake in this environment [5] and 52% of children younger than 6 years old attend CCs increases the significance of childcare institutions for nutrition intervention.

Sustainability, cost, nutrition, and health value make pulse-based foods (foods made with beans, chickpeas, peas, and lentils) a suitable dietary choice for establishing healthy eating habits among preschool children in CCs. Young children who consumed beans were found to have greater protein, fiber, potassium, and magnesium intake compared with those who did not [6]. Despite these benefits, the consumption of pulse-based foods is low in Canada [7]. Further, Canada is a global leader in pulse crop production. An increase in awareness through repeated exposure and skill in cooking pulses can potentially lead to liking of pulse-based dishes and more pulse-based dishes being offered in CCs [1,8], which can, in turn, lead to an increase in the consumption of these foods.

While much effort has been dedicated to improving fruit and vegetable intake in CCs [9-12], interventions promoting pulse-based dishes in CCs are scarce. Built on social cognitive theory and taking Piaget’s cognitive development into consideration, the Pulse Discovery Toolkit (PDTK) intervention was developed to promote consumption of pulse-based foods in CCs [13]. The PDTK is designed to offer multiple opportunities for children to taste and learn about pulses either through lessons or through pulse-based snacks and lunches. This pilot study aimed to compare knowledge, willingness to taste, and consume pulse-based foods in preschool children in 2 CCs pre- and post-PDTK intervention. In addition, nutrient contents and food group servings of the pulse-based intervention recipes were compared with the regular CC recipes. The feasibility and acceptability of the intervention from early childhood educators’ (ECE’s) and cooks’ perspectives were also assessed.

## Method

### Overview

A pre-post design pilot study with a community-based approach was carried out in 2 CCs in Saskatoon. ECEs, staff, and children in CCs were engaged in revising the PDTK before its rollout. A detailed description of the method is published by Haileslassie et al [13]; this study used a multimethod approach with both quantitative and qualitative data collection.

### Ethical Consideration

Ethical approval was obtained from the University of Saskatchewan Behavioral Research Ethics Board, and a certificate of approval was issued (Beh # 15‐388). Prior to data collection, consent and verbal assent were obtained from parents and children, respectively. As a pilot study designed to assess feasibility, 50 children were recruited from 2 CCs, a sample size consistent with recommendations in Teresi et al [14].

### The Intervention and Study Population

The PDTK was developed using principles of intervention mapping, and it was delivered over the course of 3 months in 2 CCs in Saskatoon (50 children, 8 staff) in 2016. Both centers are located in the city and provide food prepared on-site by their cook. The children in these centers are offered a morning snack, lunch, and an afternoon snack. Prior to implementation, each of the 2 facilities received copies of the PDTK manual, and ECEs and cooks received training on using the manual and the overall objectives of the project. The PDTK manual consisted of 12 lesson plans, a food service guide, 15 recipes, and 4 parent newsletters. One of the participating CCs was English-speaking, and one was French-language only. The latter required the PDTK manual to be translated into French. Trained graduate nutrition students and ECEs participated in the delivery of the lessons.

### Knowledge Assessment Questionnaire

Pre- and posttest assessment of the children’s pulse knowledge was carried out at each intervention site. A pictorial data collection questionnaire was adapted and pretested for this purpose. A similar type of pictorial tool has been previously validated and used with children [15,16].

### Plate Waste Assessment and Comparison of Nutrient Composition and Food Group Servings of Intervention and Control Recipes

The 12 weeks of the intervention were divided into 3 cycles: time-point 1 (the first 4-wk cycle), adjustment period, and time-point 2 (the last 4-wk cycle). Recipes that could possibly serve as a control for the intervention recipes were selected from the regular recipes offered in each CC (Table 1). The matching of the intervention recipes was based on their similarity to control menu items, likelihood of adaptability, and cooking time. In addition to the pulse-based foods prepared at CCs, commercially purchased products, such as puffs (extruded light and airy snacks), lentil pasta, and biscuits, were also evaluated by the children. The puff flavors were particularly developed in consultation with food science experts, educators, and children. These foods were incorporated to increase plant-based snack and meal options as opposed to the regular high-salt processed foods.

**Table 1.** Intervention recipes and their corresponding controls in the participating childcare centers in Saskatoon.

Intervention recipes	Control recipes
Lentil pizza	Mexican Pizza Chicken Pizza
Chickpea spread	Hummus Cream Cheese
Stir fry chickpea, vegetables	Stir Fry Vegetables with Beef Kidney Beans, Beef, and Vegetables
Three bean quesadillas	Chicken and Cheese Quesadillas Tuna Wrap

Plate waste measurement of the intervention and the regular recipes was taken twice during the 12-week intervention. A digital photography-enhanced application validated in previous studies captured the weight of the foods before and after consumption [17]. Food Processor Nutritional Analysis Software (Esha version 10.10.00) was used to compute the energy, nutrient breakdown (protein, total fiber, fat, saturated fat, calcium, potassium, sodium, and iron), and food group servings (meat and alternative, milk, grain, vegetable, and fruit) in order to compare the nutrient composition and food group servings of intervention and control recipes.

### Sensory Evaluation

Three types of sensory evaluation were carried out to assess the acceptability of the pulse-based intervention recipes. First, sensory evaluation was carried out as part of the lesson on 3 occasions. The children were asked to taste and report their liking using a 3-point facial hedonic scale with the options of “Yummy,” “Yucky,” and “Ok” [18]. Second, sensory evaluation of selected recipes was conducted at 2 time points to compare the degree of liking after exposure. The first was closer to the beginning of the intervention, and the second was taken at the end after several exposures to pulse-based dishes. Third, preference tasting was carried out by presenting 2 pulse-based foods at the same time and requesting the children to indicate which of the two they like the most. This was particularly used for tasting spreads, and participants were offered a choice of trying it with and without accompanying vegetables. Further details are available in the protocol by Haileslassie et al [13].

### Qualitative Data Collection: Lesson Plan Evaluation, ECE, and Cook Interviews

Weekly lesson plan evaluation using questions adapted from Sharma et al [9] was carried out at the end of each lesson. One-on-one semistructured interview guides were used to gather information from cooks and ECEs. A semistructured postintervention interview adapted from Sharma et al [19] was used to further explore the acceptability and feasibility of the PDTK educational resource by the ECEs. The cooks’ interview focused on the perceived benefits and barriers of cooking pulses and the feasibility of cooking the recipes included in the PDTK. Interviews were recorded, transcribed, and analyzed into themes [20]. The participants were given a chance to review their transcripts to validate their views or opinions.

### Data Analysis and Quality Control

To ensure a standardized procedure was followed for data collection, research assistants were trained on administering the pre-post questionnaire, conducting sensory evaluation, and plate waste measurement. Furthermore, each assistant was trained on how to instruct and prepare the participating children for sensory evaluation. A one-day training session, along with practice in a pretest CCs using data not included in the final analysis, was conducted to ensure that research assistants fully understood the data collection protocols and were proficient in applying them.

Quantitative data were analyzed using SPSS (version 24; IBM Corp; Armonk, NY, USA) and SAS (version 9.4; SAS Institute Inc. Cary, NC, USA). Results were considered statistically significant if a *P* value <.05 was obtained. SPSS was used to generate descriptive statistics and for paired comparisons. Each item on the Knowledge Assessment Questionnaire was scored as a binary categorical variable (1=correct identification; 0=incorrect identification). For each pulse item, we calculated the proportion of children answering correctly at pre- and postintervention. Chi-square and McNemar tests were used for paired nominal data to compare the pre and post pulse knowledge scores. A paired sample *t* test was also conducted on spreads (green split pea vs red bean) to determine if there was any difference between sensory acceptance at the beginning and at the end of the intervention. SAS was used to generate *t* test and generalized estimating equation (GEE) model results for plate waste measurements. The GEE model was developed to analyze the relationship between control recipes and intervention recipes, along with the co-covariates age, sex, and center across the 2 time periods (baseline and endpoint) using aggregate data. The outcome variable was the amount of food consumed, and a fixed effect was selected. The independent variables included the type of recipe (intervention vs control), time (baseline and endpoint), and covariates (age, sex, and center). The GEE model analysis is a widely used statistical model for data collection for repeated measurements, as it allows analysis for missing and unbalanced data. Since the distribution of all responses (outcomes) is from the gamma distribution family, we were required to choose gamma distribution and “power (-1)” as link function for conducting “pro Genmod” in SAS. While the GEE model accounts for missing data, a sensitivity analysis was not conducted. Using aggregate data, an independent *t* test was performed to compare the mean consumption and the mean consumption proportion of both the intervention and control recipes. Consumption proportion (proportion of food consumed to food served) was calculated for each intervention and control recipe using the formula provided below:


$$Consumption\;Proportion=\frac{\begin{array}{c} sum\;of\;the\;amount\;of\;all\\ \;intervention\;(or\;control\;foods)\;a\;specific\;child\;eats \end{array}}{\begin{array}{c} sum\;of\;the\;amount\;of\;intervention\;foods\\ \;(or\;control\;foods)\;that\;was\;given\;to\;that\;specific\;child \end{array}}$$

The qualitative data analysis followed thematic analysis, consistent with the coding reliability approach described by Braun and Clarke [21]. The validated transcripts from ECE and cook interviews were analyzed separately to reflect their distinct perspectives. In the first phase, transcripts were reviewed line by line to identify recurring ideas and patterns. In the second phase, initial codes were independently reviewed by a second researcher to verify consistency and refine theme development. In the third phase, emerging themes were discussed collaboratively to ensure coherence and resolve discrepancies through consensus. Relevant participant quotes were organized under final thematic categories to illustrate and support the findings. To enhance trustworthiness and rigor, the team used researcher triangulation, peer debriefing, and an audit process. Themes were refined iteratively to ensure they accurately represented the data.

### Ethical Consideration

Ethical approval was obtained from the University of Saskatchewan Behavioral Research Ethics Board. Prior to data collection, consent and verbal assent were obtained from parents and children, respectively.

## Result

### Overview

A total of 50 children between the ages of 2 and 5 years from 2 CCs participated in the study. All ECEs (n=5) from both schools completed the lesson plan evaluation forms. In addition, the ECEs and cooks in each center participated in a postintervention interview.

### Pre and PostIntervention Pulse Knowledge of Preschool Children

Pre and postintervention pulse knowledge of the preschool children is presented in Table 2. No statistically significant improvements in children’s pulse knowledge from pre to postintervention across all items were noted, though the increase in correct identification of chickpeas approached significance (*P*=.07).

**Table 2.** Pre and postintervention pulse knowledge of preschool children in 2 childcare centers in Saskatoon (n=21). The number of children who have a complete set of pre-post knowledge assessment data in a McNemar’s test.

Questions	Pretest, n (%)		Posttest, n (%)		*P* value
	Correct	Incorrect	Correct	Incorrect	
Distinguishing between lentils and beans	14 (67)	7 (33)	13 (62)	8 (38)	≥.99
Identification of chickpeas	2 (10)	19 (91)	7 (33)	14 (67)	.07
Identification of lentils	1 (5)	20 (95)	2 (10)	19 (90)	≥.99
Identification of beans	8 (38)	13 (62)	11 (52)	10 (48)	.68
Identification of peas	6 (27)	15 (71)	8 (38)	13 (62)	.61

### Plate Waste Measurement of Pulse-Based Foods

The amount of food consumed from the intervention and the control recipes at time-point 1 and time-point 2 is described in Table 3. Significant differences in mean consumption between the control and intervention recipes were noted among most of the variables at both times, except for the fruit and vegetable servings, meat and alternative servings, and fiber content. The GEE Model analysis showed significantly lower mean consumptions in time-point 2 for all the variables of interest, except for the fruit and vegetable servings. The mean consumption proportion from the intervention and the control recipes at time-point 1 and time-point 2 is presented in Table 4. At time-point 1, no significant difference was observed in the mean consumption proportions of all the nutrients of interest between the intervention and control recipes, except for the higher saturated fat from the control recipes and the higher trans-fat from the intervention recipes. At time-point 2, significant differences were only observed between the mean consumption proportion of the quantity (*P*=.049) and the protein content (*P*=.04); both of which were higher among the control recipes in comparison to the intervention recipes. In terms of the consumption proportion, the GEE model revealed significant differences in the total quantity and most nutrients, except for the servings of fruit and vegetables, meat, whole grain, trans-fat, and sugar content.

**Table 3.** The quantity, food groups serving, and nutrient contents of intervention and control recipes consumed by preschool children in 2 childcare centers at time-point 1 and time-point 2 during a 12-week pulse discovery tool kit intervention.

	Time-point 1				Time-point 2				GEE^a^
	Control (n=39), mean (SD)	Intervention (n=38), mean (SD)	Mean difference^b^	*P* value^c^	Control (n=37), mean (SD)	Intervention (n=27), mean (SD)	Mean Difference^b^	*P* value^c^	*P* value
Quantity (g)	293.5 (172.7)	211.6 (157.9)	81.97	.03	178.5 (148.0)	108.8 (114.2)	69.63	.049	<.001
Fruit and vegetables serving	0.95 (0.95)	0.96 (0.76)	−0.01	.95	0.42 (0.49)	0.46 (0.61)	−0.04	.77	.21
Meat and alternative serving	0.98 (0.54)	0.74 (0.59)	0.24	.06	0.62 (0.50)	0.40 (0.44)	0.22	.06	.001
Whole grain serving	39.10 (25.11)	21.55 (20.78)	17.54	<.001	28.96 (24.92)	4.67 (8.40)	24.28	<.001	<.001
Protein (g)	30.82 (15.61)	20.24 (14.79)	10.58	.003	21.64 (15.61)	9.50 (10.25)	12.13	<.001	<.001
Fiber (g)	5.92 (3.40)	5.24 (4.00)	0.68	.42	3.79 (3.49)	2.16 (2.32)	1.62	.04	.02
Carbohydrates (g)	56.50 (38.75)	36.02 (27.27)	20.48	.01	38.94 (37.90)	19.03 (17.89)	19.91	.02	<.001
Calories (kcals)	554.3 (303.5)	316.8 (236.6)	237.5	<.001	387.4 (296.6)	155.1 (152.3)	232.2	<.001	<.001
Sodium (mg)	1255.1 (882.9)	531.0 (405.8)	724.1	<.001	624.5 (499.5)	285.5 (276.7)	339.0	.002	<.001
Saturated fat (g)	9.18 (4.75)	4.73 (4.06)	4.45	<.001	6.60 (4.51)	2.05 (2.64)	4.55	<.001	<.001
Trans fat (g)	0.32 (0.35)	0.005 (0.01)	0.31	<.001	0.26 (0.30)	0	0.26	<.001	.01
Sugar (g)	4.38 (2.79)	3.18 (2.39)	1.20	.049	2.85 (2.78)	1.56 (1.67)	1.28	.04	<.001

a GEE: generalized estimating equation.

b Note-mean differences: subtracting means of control variables from means of intervention variables.

c Independent *t* test.

**Table 4.** The consumption proportion (Consumption proportion=sum of the amount of all intervention (or control foods) a specific child eats sum of the amount of intervention foods (or control foods) that was given to that specific child) of intervention and control recipes at time-point 1 and time-point 2 during a 12-week pulse discovery toolkit intervention in 2 childcare centers in Saskatoon.

	Time-point 1				Time-point 2				GEE^a^
	Control (n=39), mean (SD)	Intervention (n=38), mean (SD)	Mean difference^b^	*P* value^c^	Control (n=37), mean (SD)	Intervention (n=27), mean (SD)	Mean difference^b^	*P* value^c^	*P* value
Quantity (g)	0.79 (0.23)	0.73 (0.28)	0.06	.27	0.86 (0.20)	0.73 (0.30)	0.13	.05	.001
Fruit and vegetables servings	0.78 (0.25)	0.74 (0.29)^d^	0.04	.56	0.81 (0.21)^e^	0.68 (0.35)	0.13	.09	.08
Meat servings	0.76 (0.27)	0.74 (0.27)	0.02	.77	0.82 (0.24)	0.73 (0.30)	0.10	.14	.09
Whole grain servings	0.74 (0.33)	0.68 (0.37)^f^	0.06	.51	0.82 (0.29)	0.87 (0.29)	−0.05	.65	.08
Protein (g)	0.80 (0.23)	0.73 (0.28)	0.07	.28	0.85 (0.21)	0.72 (0.30)	0.13	.04	<.001
Fiber (g)	0.78 (0.26)	0.70 (0.31)	0.08	.24	0.85 (0.21)	0.73 (0.31)	0.12	.07	.003
Carbohydrates (g)	0.81 (0.22)	0.71 (0.29)	0.09	.13	0.87 (0.21)	0.76 (0.30)	0.11	.09	.002
Calories (kcals)	0.80 (0.22)	0.71 (0.29)	0.09	.15	0.86 (0.20)	0.74 (0.29)	0.12	.07	<.001
Sodium (mg)	0.79 (0.22)	0.72 (0.29)	0.07	.23	0.86 (0.20)	0.75 (0.29)	0.11	.09	.005
Saturated Fat (g)	0.82 (0.19)	0.67 (0.3)	0.15	.02	0.86 (0.20)	0.75 (0.31)	0.12	.08	<.001
Trans fat (g)	0.76 (0.30)	1.00 (0)^g^	−0.24	.03	0.85 (0.26)	—^h^	—	—	.22
Sugar (g)	0.78 (0.24)	0.72 (0.29)	0.07	.25	0.85 (0.21)	0.73 (0.31)	0.12	.06	.001

a GEE: generalized estimating equation.

b Mean differences: subtracting means of control variables from means of intervention variables.

c Independent *t* test.

d Intervention recipes (n=36).

e Control (n=28) and intervention recipes (n=26).

f Intervention recipes (n=34).

g Intervention recipes (n=8).

h Not applicable.

### Sensory Evaluation of Pulse-Based Foods

The results obtained from sensory evaluation of lentil pasta, refried bean wrap, lentil smoothie, and 2 types of extruded snacks (puffs) are presented in Table 5. Majority (92% and 72%) of the children rated the refried bean wrap and lentil smoothie “yummy,” respectively. Some children (9.4%, 17%, and 15%) refused to taste the lentil smoothie, red pepper and lime flavor puff, and white cheddar puff, respectively.

**Table 5.** Sensory evaluation of selected pulse-based foods by preschool children during pulse discovery toolkit intervention in 2 childcare centers in Saskatoon.

Pulse-Based foods	Hedonic rating^a^			
Yummy, n (%)	OK, n (%)	Yucky, n (%)	Not willing to taste, n (%)
Lentil pasta (n=17)	10 (59)	4 (24)	3 (17.6)	—^b^
Refried bean wrap (n=25)	23 (92)	1 (4)	1 (4)	—
Lentil smoothie (n=32)	23 (72)	2 (6)	4 (12.5)	3 (9.4)
Red pepper and lime flavor puff (n=12)	8 (67)	0 (0)	2 (17)	2 (17)
White cheddar flavor puff (n=13)	6 (46)	4 (31)	1 (8)	2 (15)

a A 3-point facial hedonic scale with ratings of “Yummy,” “Yucky,” and “Ok”.

b Not applicable.

### Comparison of the Nutrient Composition Between Intervention and Control Recipes

The nutrient composition of intervention and control recipes is provided in Table 6. The comparison revealed higher energy, lower fat, and lower sodium content in most of the intervention recipes.

**Table 6.** The nutrient composition of 100 g of the pulse discovery toolkit intervention and the control recipes from 2 childcare centers in Saskatoon.

Recipes	Energy (Kcals)	Protein (g)	Total fiber (g)	Fat (g)	Saturated fat (g)	Ca (mg)	K (mg)	Na (mg)	Iron (mg)	Meat svgs^a^	Milk svgs	Grain svgs	Veg/fr^b^ svgs
Mexican pizza (R)^c^	134.58	8.17	4.22	3.04	1.3	190.02	285.44	460.52	1.59	0.1	0.22	0.63	0.16
Lentil pizza (I)	141.64	8.84	2.86	5.87	3.34	143.1	254.47	186.46	1.19	0.21	0.21	0.20	0.40
Chicken pizza (R)^d^	206.69	10.37	1.6	6.19	3.25	148.88	197.78	362.92	1.61	0.10	0.30	1.22	0.39
Lentil pizza (I)	142.83	8.78	2.55	5.64	3.29	144.17	247.19	211.21	1.71	0.21	0.40	0.21	0.40
Hummus and tortilla (R)	245.92	8.68	4.36	3.04	1.3	78.92	34.58	474.99	2.27	0.30	0	1.90	0
Chickpea spread and tortilla (I)	243.3	8.61	4.42	5.24	3.34	80.16	39.38	503.57	2.28	0.30	0	1.90	0.03
Pita with cream cheese (R)	297.03	8.06	1.48	12.07	6.46	89.95	125.92	465.3	1.88	0.30	0	1.90	0
Pita with chickpea spread (I)	171.13	6.84	0.67	3.23	0.37	53.64	117.17	453.52	1.33	0.30	0	1.90	0.03
Stir fry vegetables with beef (R)	202.12	14.58	0.42	14.51	4.45	22.51	257.86	309.5	1.15	0.30	0	1.90	0
Stir fry chickpea, vegetables, and beef (I)	109.77	11.45	1.27	4.43	1.42	27.56	253.61	145.53	1.35	0.30	0	1.90	0.03
Kidney beans, beef, and vegetable (R)	136.13	12.09	1.94	6.62	2.34	32.97	278.07	145.15	1.48	0.72	0	0	0.34
Stir fried chicken, chickpea, and vegetable (I)	86.32	10.7	1.27	1.87	0.27	29.36	238.72	131.19	0.78	0.50	0	0	0.96
Chicken and cheese quesadillas (R)	273.02	20.64	1.55	15.74	8.33	270.87	132.39	375.94	1.4	0.53	0.70	0.70	0
Three bean quesadillas	231.69	10.63	4.46	9.37	4.04	166.72	132.06	353.46	2.06	0.30	0.31	1.09	0.10
Tuna wrap (R)	228.53	10.87	3.72	8.15	1.65	53.56	78.03	422.7	1.87	0.30	0	1.45	0.21
Three bean quesadillas (I)	219.54	10.89	4.64	8.07	3.92	162.67	132.06	364.57	2.14	0.30	0.31	1.09	0.10

a Svgs: servings.

b Veg/fr: vegetable and fruit.

c *R*: Regular Recipe.

d I: Intervention recipe.

### Qualitative Findings: Educator and Cook Perspectives on the PDTK Intervention

The qualitative data are presented in three distinct sections: (1) open-ended lesson plan evaluations, (2) ECE interviews, and (3) cook interviews. These are presented separately to reflect the distinct data collection and analysis processes used for each group.

### Lesson Plan Evaluations

ECEs rated the clarity of the instructions of most lessons as “good” on a 5-point scale that ranges from poor, fair, good, very good, to excellent. They indicated that most of the learning objectives were appropriate for this age group. However, ECEs also reported that some of the activities were difficult for the children in this age group to comprehend. One of the ECEs mentioned particularly in lesson 12 (mystery bucket).

*I think it was difficult for children to identify food groups*.

The ECEs also noted children’s reactions to the lessons and reported that most children were engaged in the PDTK activities. Examples of responses are as follows:

*They had smiles on their faces as they were eager to answer the questions correctly to show what they already knew.*

[Lesson 1- Healthy Eating]

*They were engaged and enthusiastic and seemed to have fun.*

[Lesson 2- Meet the pulses]

*Dramatic play is one of the children’s favorite games. They couldn’t wait to have a turn being a server, customer or cook*.

[Lesson 10- Let’s Play Restaurant]

*They loved it. They concentrated on their menus and had a lot of fun playing restaurant.*

[Lesson 10 - Let’s Play Restaurant]

Recommendations on improving the acceptability and feasibility of these lesson plans included breaking down the lesson plan activities into smaller components, as well as dividing the children into smaller groups to reduce wait times and the risk of loss of interest. The following quotes are responses related to the acceptability of PDTK activities for implementation in their CCs.

*Yes, anything promoting active lifestyle is always acceptable*.

[Lesson 9 Pulse Bowling]

*Yes, having children be part of the meal preparation encourages healthy eating.*

[Lesson 10 - Let’s Make Our Own Food]

*Yes, we can insert [the program] into the curriculum for the health of the Canadian children and to avoid obesity in children.*

[Lesson 11- Let’s play restaurant]

### Educators’ Perception of the PDTK

Key themes were collated from ECE responses about their PDTK experiences. The primary themes identified from the interviews were as follows: (1) feasibility and acceptability of the PDTK, (2) barriers to implementation, and (3) recommendations for the PDTK. Table 7 provides a snapshot of themes developed and examples of corresponding quotes from the educators’ observations and experiences throughout the intervention.

**Table 7.** A snapshot of the themes and corresponding quotes identified from interviews.

Themes	Quotes
1.Feasibility and acceptability of the PDTK	*Since you guys started, I found every day was a positive influence on the kids. I really enjoyed that you guys came and did these. We don’t often have the opportunity to have something like this to encourage healthy eating. We do it on our own and make up our own. So, to have someone come in that already has something set up and in place was awesome, because I love encouraging kids to eat healthy food.* [ECE # 1] *For this place, the people are not moving too much and the kids are not moving too much. It is not like, I came from [name of country] and the kids they can walk and we don’t have a problem with winter. They stay at home and they are not moving. Sometimes I see what there are eating too much is giving them obesity. But, when you have food that control it. Like your program it will help them to stay healthy. It will help them. I would like to put it that way. If I had my own center, like this I would put in my own program as quickly.* [ECE #2] *I think we can try some activities. So, for example, we made taco with beans and I think it is a good curriculum for children. Because, the activities included sensory activities. So we could taste it, smell it and feel. All sensory were included in these activities. Also, children they are interested in making food and touching something. So, we can do them next time*. [ECE #3] *They were engaged because sometimes they would ask normally when you people are coming…even the kids would ask, what they are going to give us to eat today. They will ask like that or maybe they will ask … (the cook)…. what are you going to cook for us today? Beans and what, they need to know.* [ECE #3]
2.Barriers to implementation	*Not, that I didn’t enjoy that is not the word. I think some activities were very, not difficult but were not really appropriate for their age, is appropriate the word? (Yea). They were very young for the kind of activities. It wasn’t really for them. It wasn’t really helpful.* [ECE #1] *don’t think it’s important to know about food groups and guide. We don’t need for children. We just can explain that milk is for teeth and meat is for our health and just explain simply and they can accept. If we added some information it it’s for first group and some food include in the first group….. They are just 2 and half years old, it’s difficult for them.* [ECE #2] *Actually, we didn’t have time to do the activity. We have 20 children and plus we have two teachers with the 20 children. For me I think it was difficult to do these lessons. Because, before starting these activities we need to explain what we are going to do and what kind of things I do with some stuff. But, actually, sometimes when I was alone with many children it was so difficult.* [ECE #3]
3. Recommendation for the PDTK	*What I enjoyed was when they had to see the different kind of peas, the different colors and when we had to do the smoothies. Because they have to count, measure and they have to taste, they have to touch. It was really physical; you know sensory for them and when they have to grow all the seeds.* [ECE #4] *We need more big charts, big pictures, not just the small paper to show exactly what it actually is. Also, we need, more time to explain what it is. So, I prefer, one day we can do about the first food group and the next day the second group ………* [ECE #5]

### Recommendations for Enhancing the PDTK Resource

The educators had several suggestions for strengthening the sustainability of the PDTK resource. These include (1) more training for ECE; (2) separate group lesson plan activities according to specific age groups; (3) introduction of concepts one at a time, thus breaking the lesson plan into smaller components; (4) large demonstration charts to increase the level of engagement; and (5) constant repetition of the pulse-related concepts. It was also discussed that the educator’s level of interest and level of experience were key to increasing buy-in within centers. Overall, educators perceived that the PDTK has significant potential in increasing acceptance of novel food at an early age. One of the ECE’s stated,

*It’s awesome. It’s exactly what Saskatchewan needs right now. More people are advocating and starting young.*

[ECE # 2]

### Cooks’ Perceptions of the PDTK Recipes

The cooks in both CCs indicated they had prepared at least one pulse-based food every 2 weeks prior to the intervention. Neither cook saw any barrier to cooking pulses in their facility. When asked about opportunities for including more pulse-based food products, they indicated that having them on their menus and keeping cooked pulses in the freezer would increase their use in regular dishes. They indicated that it is feasible to include recipes from the PDTK resource in their menus. One of the cooks mentioned that the nutrition intervention provided more opportunities and experience in cooking new pulse recipes. They reported that most of the recipes are easy to follow and to shop for, and the food tastes good. One of the cooks indicated that she liked the lentil pizza as the children enjoyed it, and it was good for the children’s health. She further mentioned that this dish can be added to their menu. The 3-bean quesadilla recipe was the favorite of one of the cooks. She said it was fun to make, whereas the other cook said that, while it was a good recipe, it was hard to cook. They both believed that the children were receptive to the recipes that they tried. Overall, they both said that they liked all the recipes, with some needing modifications.

## Discussion

### Principal Findings

This study presents findings from Phase 2 of a 3-phase project evaluating PDTK, a 12-week multicomponent nutrition intervention designed to promote consumption of pulse-based foods among preschool children in CCs. As outlined in the protocol [13], Phase 2 was conducted as a pilot study with a primary focus on assessing implementation processes and determining whether the intervention could be delivered as intended within real-world childcare settings. Accordingly, feasibility was operationalized through process-oriented outcomes, including cooks’ and educators’ perceptions of PDTK recipes, lesson plan evaluations, and recipe tasting activities, with particular emphasis on the practicality of recipe preparation and alignment with existing curricula. The impact of the intervention on knowledge, acceptability, and consumption of pulse-based foods was assessed. Feasibility and acceptability of the PDTK intervention for implementation in CCs were captured through lesson plan evaluations and interviews with ECEs and cooks.

To our knowledge, no recent studies have specifically examined pulse consumption in childcare settings for this age group in Canada. While the data for our study were collected a few years ago, it remains pertinent given the lack of significant interventions or policy changes that could have altered pulse consumption trends. The only notable changes are the release of the new Canada’s Food Guide in 2019 and the increased promotion of plant-based diets. Evidence from a study by Hank et al [22] explored the potential impact of the 2019 Canada’s Food Guide on the eating environments and food provided in early learning and CCs across Canada. This study found that representatives from CCs faced challenges in interpreting and implementing the new guidelines. As a result, no significant changes to children’s dietary patterns in these settings were observed. This suggests that, despite the shift in dietary recommendations, pulse consumption in childcare environments has likely remained stable. While isolated case studies of transitions toward plant-based menus [23] have been reported in Canadian CCs, these appear to be the exception rather than the norm. Recent studies in Canada further reinforce the importance of childcare-based interventions in shaping children’s dietary behaviors and daily nutrient intake [24,25].

Although some improvement was noted in the percentage of preschool children who correctly identified pulses in this study, the improvement was not noted for all pulses. This could be partly explained by the longer duration of some lessons, preschoolers’ short attention span, and the small sample size. These findings contradict similar studies that introduced other food items, such as fruits and vegetables, to preschoolers [26,27]. Many of these studies indicated a significant increase in knowledge of fruit and vegetables between the pre and posttests. The differences in the findings between this pulse intervention and the fruit and vegetable interventions could be attributed to the familiarity of households with fruit and vegetables or the novelty of exposing young children to pulses.

A significantly greater amount of the regular recipes was consumed at time-point 1 than the intervention recipes; however, this difference was not seen when the consumption proportion was considered. Unlike the finding at time-point 1, the children consumed a higher quantity of the regular recipes than the intervention recipe at time-point 2, even when the consumption proportion was considered. These results contradict findings of increased consumption of lentils after repeated exposure [1]. In contrast to our study, the children in Ramsay et al [1] study were exposed to lentils 1 to 2 times per week for a total of 13 weeks. Though the children in our study were exposed to different pulse-based foods at least twice a week, the same dish was only offered every 3 weeks. In addition, Ramsay et al [1] let the children taste unseasoned lentils, while our study exposed children to a variety of pulse-based foods. These findings highlight the possibility of higher effectiveness of repeatedly exposing to one specific pulse-based dish at a time than exposure to a series of pulse-based dishes to increase intake and acceptability of pulses among young children. In addition, the pulse-based dishes introduced in this study were mixed dishes, unlike most of the studies [28,29] that reported a significant increase in acceptability and intake after repeated exposure to food. The use of mixed dishes in PDTK may have limited the positive impact of the repeated exposure on the intake and acceptability of the pulse-based dishes in our study.

The comparison between the intervention and regular recipes, which revealed lower total kilocalories and fat content in most of the intervention dishes, is not surprising given the low energy, fat, and sodium contents of pulses [30-32]. In addition, most of the regular recipes selected for comparison contain animal source foods, which could contribute to the higher fat and energy content of the regular recipes.

Unsurprisingly, some children were not willing to taste the pulse-based dishes offered to them during the sensory evaluation activities. Similar reactions have been reported before, where novel food products served to children were rejected, a phenomenon described as neophobia, which refers to the fear or avoidance of new or unfamiliar foods [33]. The children in our study were at the peak age of neophobia [34]. Food reluctance has been reported as a key challenge to offering plant-based meals in Canadian CCs [22], and food neophobia remains a well-documented barrier in early childhood nutrition, as highlighted in a recent multicountry systematic review demonstrating that neophobia contributes to children’s reluctance to try unfamiliar foods and limits dietary variety [35]. The recipes with the highest rating of “Yummy,” refried bean wrap and lentil smoothie, were interestingly recipes that children took part in preparing as part of the lesson activities. Similarly, though the study population was older children (5‐7 y-old) and the finding could not solely be linked to their participation in the preparation, children rated foods they prepared “more yummy” than foods served to them [36].

Findings from ECE semistructured interviews and the lesson plan evaluations revealed that the ECEs reacted favorably to the curriculum, especially to lesson plan activities that had both sensory and hands-on components. The ECEs stated that the children particularly enjoyed preparing the snacks, role-playing, and gardening, and were highly engaged when performing these activities. The educators in this study also discussed a few factors that could potentially act as barriers to the feasibility and acceptability of the nutrition curriculum. Key barriers identified were the length of time to complete each activity and the suitability of some of the activities to meet the children’s cognitive level. These findings are similar to a pilot study conducted by Sharma et al [9], a healthy nutrition and a physical activity program in 2 CCs.

In our study, the cooks from the participating CCs did not report any barriers to cooking pulses in their facility, contrary to a previous study that examined the barriers and opportunities to serving pulses in school meals, which includes time for cooking dry beans [37]. In our study, the PDTK recipes use canned pulses or dry lentils, which do not take as long as dry beans to cook. However, the need for modification to make the recipes easier to cook in childcare settings was noted in our study.

To better suit different developmental stages of preschool children, the intervention’s lesson plans could be adapted by varying the complexity of activities based on cognitive and motor skills. For instance, in Lesson One, “Naming Pulses,” the lesson could be tailored as follows: For younger preschoolers (ages 3‐4 years), the lesson plan can focus on sensory exploration, where children touch and sort different pulses (eg, beans, lentils, peas) by size or color, enhancing their fine motor skills and hand-eye coordination. Teachers can also encourage language development by labeling the pulses and asking children to repeat their names, introducing basic vocabulary. For older preschoolers (ages 4‐5 years), the activity can include problem-solving tasks such as sorting pulses by categories or counting them, along with more precise motor tasks like transferring pulses using scoops, while also fostering cognitive skills through matching games. Though our study looked at specific pulse-based dishes, we can speculate that including a wider variety of pulse-based recipes in the intervention could increase its appeal and effectiveness for children by offering more fun, diverse, and flavorful options that cater to different tastes and preferences, making the learning experience more engaging. The results were instrumental in guiding the subsequent roll-out of the intervention in phase 3 in 4 CCs. Revisions include lesson plan modifications to better align with developmental stages and age-appropriate learning, and simplified activities for younger children to emphasize sensory exploration and basic identification tasks, rather than more complex components that require higher levels of cognitive processing. These findings informed practical adjustments to the intervention design, recipe selection, and PDTK lesson plans. The lessons learned were also used to expand the program to elementary school [38]. The growing interest in plant-based dietary patterns for both health and environmental sustainability further underscores the continued relevance of interventions such as the PDTK. The growing interest in plant-based dietary patterns for both health and environmental sustainability further underscores the continued relevance of interventions such as the PDTK, particularly in light of Canadian evidence demonstrating both the feasibility of plant-based menu implementation in childcare settings and the significant role these environments play in shaping children’s dietary behaviors [24,25].

### Limitations

First, due to the convenience sampling technique used and the small sample size, the generalization of the findings is limited. Second, the 2 CCs have different meal practices; in one of the centers, children are encouraged to portion their own food, whereas in the other center, food is served prior to mealtime. Additionally, children not attending all sessions during data collection or when lessons are offered may have reduced the impact of the PDTK intervention. Lack of control groups limited the study’s ability to draw strong causal inferences, and future studies with a control group will better isolate the intervention’s effects by comparing recipients to nonrecipients. The inclusion of cooks was limited to those working at the center, which resulted in a smaller sample size.

### Conclusions

The PDTK intervention resulted in an increase in the percentage of preschool children who correctly identified pulses, but not their intake of pulse-based foods. The findings from the pilot testing also revealed that modifications are necessary to make some of the lessons more age-appropriate. Cooks found most of the PDTK pulse-based recipes to be acceptable and were willing to include them in the centers’ menus while noting that some need adjustments to make them easier for batch cooking. Further research may be necessary to determine what effects a modified PDTK intervention will have on preschool children’s pulse knowledge, acceptability, and intake of the intervention pulse-based foods. In addition, a follow-up assessment can determine if behavioral changes are sustained, the durability of the intervention’s effects, and whether further support is needed for long-term sustainability.

## Abbreviations

CC: childcare center
ECE: early childhood educator
GEE: generalized estimating equation
PDTK: Pulse Discovery Toolkit
